# Neuronal extracellular signal-regulated kinase (ERK) activity as marker and mediator of alcohol and opioid dependence

**DOI:** 10.3389/fnint.2014.00024

**Published:** 2014-03-11

**Authors:** Eva R. Zamora-Martinez, Scott Edwards

**Affiliations:** ^1^Department of Molecular and Cellular Neuroscience, The Scripps Research InstituteLa Jolla, CA, USA; ^2^Department of Physiology and Alcohol and Drug Abuse Center of Excellence, Louisiana State University Health Sciences CenterNew Orleans, LA, USA

**Keywords:** addiction, amygdala, drug dependence, extracellular signal-regulated kinase, nucleus accumbens, protein phosphorylation, reward, withdrawal

## Abstract

Early pioneering work in the field of biochemistry identified phosphorylation as a crucial post-translational modification of proteins with the ability to both indicate and arbitrate complex physiological processes. More recent investigations have functionally linked phosphorylation of extracellular signal-regulated kinase (ERK) to a variety of neurophysiological mechanisms ranging from acute neurotransmitter action to long-term gene expression. ERK phosphorylation serves as an intracellular bridging mechanism that facilitates neuronal communication and plasticity. Drugs of abuse, including alcohol and opioids, act as artificial yet powerful rewards that impinge upon natural reinforcement processes critical for survival. The graded progression from initial exposure to addiction (or substance dependence) is believed to result from drug- and drug context-induced adaptations in neuronal signaling processes across brain reward and stress circuits following excessive drug use. In this regard, commonly abused drugs as well as drug-associated experiences are capable of modifying the phosphorylation of ERK within central reinforcement systems. In addition, chronic drug and alcohol exposure may drive ERK-regulated epigenetic and structural alterations that underlie a long-term propensity for escalating drug use. Under the influence of such a neurobiological vulnerability, encountering drug-associated cues and contexts can produce subsequent alterations in ERK signaling that drive relapse to drug and alcohol seeking. Current studies are determining precisely which molecular and regional ERK phosphorylation-associated events contribute to the addiction process, as well as which neuroadaptations need to be targeted in order to return dependent individuals to a healthy state.

## Introduction

Drugs of abuse act upon a variety of brain centers to alter neurotransmission and intracellular signaling cascades. With the perplexing abundance of drug-induced neuroadaptations, the question remains which alterations result from simple pharmacological mechanisms following drug exposure, and which changes go on to drive the aberrant behavioral mechanisms responsible for addiction. This dilemma has led to multiple conceptualizations of the neurophysiological mechanisms underlying the graded transition to addiction. Some have noted the remarkable persistence of relapse propensity during periods of attempted abstinence, characterizing addiction as a disorder of learning and memory (Kelley, 2004; Hyman, 2005; Hyman et al., 2006). Within this model, drugs of abuse act as unconditioned stimuli that usurp brain reinforcement circuitry to focus reward-related pursuits away from natural or adaptive rewards toward pursuit of the drug of choice (Kalivas and Volkow, 2005). Such a narrowing of behavioral repertoire is driven by highly incentivized drug-related cues and contexts that have been repeatedly paired with drug experiences (Self and Nestler, 1998; Kalivas and O’brien, 2008). Others have highlighted the engagement and potentiation of brain stress (or anti-reward) circuitry that follows particularly excessive or escalated drug intake observed in addicted populations (Ahmed and Koob, 1998). The gradual transition from limited drug sampling to compulsive intake may actually represent a substantial interaction of both within- and between-system neuroadaptations across brain reinforcement circuitry that contributes to the persistence of the addicted phenotype (Edwards and Koob, 2010).

With the overwhelming plasticity that has been observed in preclinical models of reward and addiction (Edwards and Koob, 2012), neuroscientists have long-searched for the most important molecular substrates for purposes of disease conceptualization and therapeutic targeting (Kalivas and Volkow, 2005; Nestler, 2005). In 2007, Girault and colleagues proposed that extracellular signal-regulated kinase (ERK1/2), specifically the ERK2 isoform, acts as an intracellular interface for signaling the intersection of drug reward and drug-related contextual information (Girault et al., 2007). Their conceptualization focused on the convergence of dopamine and glutamate transmission within medium spiny neurons of the striatum (Valjent et al., 2005; Philibin et al., 2011), although similar signaling convergences could involve a variety of neurotransmitters at a number of critical limbic centers involved in various stages of the addiction process. The two major ERK isoforms are present in multiple brain regions, yet exhibit a noticeable rostral-caudal gradient whereby ERK1 levels increase and ERK2 levels decrease from rostral (frontal cortex ERK1/ERK2 ratio of 0.16) to caudal (pons/medulla ERK1/ERK2 ratio of 1.5) regions (Ortiz et al., 1995). ERK1/2 activation is predicated on phosphorylation of neighboring tyrosine and threonine residues (Robbins et al., 1993) by an upstream ERK kinase (or MEK; Payne et al., 1991) in response to a wide array of extracellular signals. Pharmacological inhibition of MEK has implicated central ERK activity in a range of neurobiological phenomenon, including fear conditioning (Sananbenesi et al., 2003), affective behavior (Einat et al., 2003), and central pain processing (Fu et al., 2008; Cao et al., 2009). Moreover, recent data have implicated ERK phosphorylation and activity across multiple brain regions throughout various stages of the addiction timeline. The current review focuses on the critical role of ERK signaling in producing dependence on alcohol and opioids, two widely abused substance classes (Substance Abuse and Mental Health Services Administration, 2008). For an excellent overview of the role of ERK in cocaine addition, see Lu et al. (2006).

**Figure 1:**
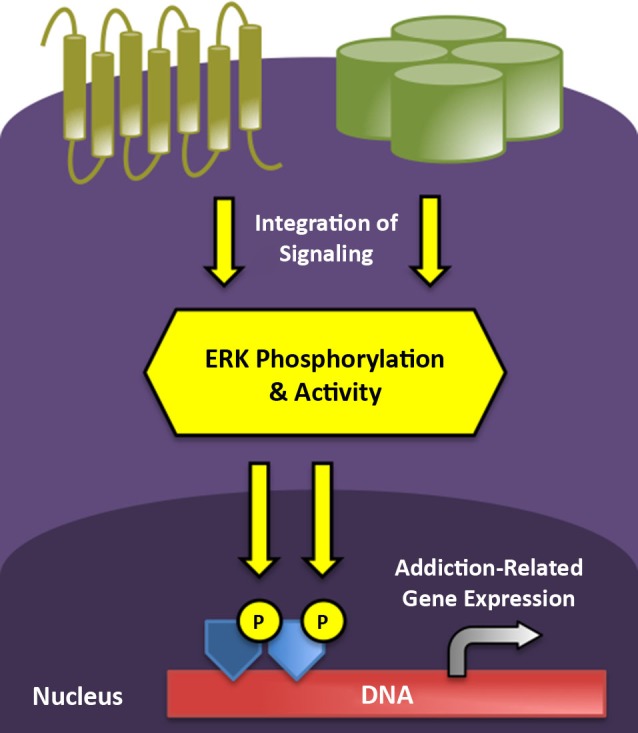
**ERK phosphorylation integrates and impacts multiple levels of intracellular signaling to mediate short- and long-term neuroplasticity associated with the transition to addicted states**. G protein-coupled receptors (gold) and ion channels (green) represent two major receptor classes that modify ERK phosphorylation in stress and reward circuitry following neurotransmitter binding. Active ERK can translocate to the nucleus to drive addiction-related patterns of gene expression following the subsequent phosphorylation of transcription factors (dark blue) and core histones (light blue).

As to how transient ERK phosphorylation might mediate the chronic, relapsing disorder of addiction, it has been demonstrated that ERK/mitogen-activated protein kinase (MAPK) activation often leads to more stable changes in gene expression via modification of the post-translational properties of histone proteins (Day and Sweatt, 2011). Histones are nuclear proteins tightly and ionically linked with DNA, normally serving to silence gene transcription. Modifications of histone proteins alter their association with DNA, rendering genetic material more or less accessible to transcriptional machinery (Cheung et al., 2000). As one example of this signaling cascade, in striatal neurons, ERK is capable of activating mitogen- and stress-activated kinase 1 (MSK1), which phosphorylates histone H3, leading to the induction of the immediate early genes c-Fos and c-Jun (Brami-Cherrier et al., 2007). Consequently, repeated activation of the ERK cascade by drugs of abuse or environmental stimuli may promote long-term gene expression and neuronal plasticity associated with the persistence of addiction via such epigenetic modifications (Figure 1). In the following, we provide an overview of the role of ERK activity in the addiction process, from the initial actions of acute alcohol and opioid exposure to the effects of cues and contexts that characterize addiction as a chronic, relapsing disease.

## Effects of alcohol exposure on neuronal extracellular signal-regulated kinase (ERK) phosphorylation

The role of the ERK signaling pathway in mediating ethanol sensitivity has been demonstrated in a variety of species, including zebrafish (Peng et al., 2009) and drosophila (Corl et al., 2009; Eddison et al., 2011). For example, ethanol acts through the ERK pathway to promote the adaptive camouflage response in zebrafish via corticotropin-releasing factor (CRF; Wagle et al., 2011). Ethanol impacts ERK/MAPK signaling across multiple organ systems (Aroor and Shukla, 2004), highlighting the central role of alcohol in human health and disease processes (National Institute on Alcohol Abuse and Alcoholism, 2010). Soon (15 min) after a 1 g/kg intragastric or intraperitoneal administration of alcohol to rats, neuronal ERK phosphorylation is increased in extended amygdala regions (Ibba et al., 2009; Thorsell et al., 2013), including the nucleus accumbens, bed nucleus of the stria terminalis, and central amygdala. This time point corresponds to peak ethanol (Quertemont et al., 2003) and dopamine (Melis et al., 2007) levels after administration of this dose, and also produces a conditioned place preference (CPP) in male (Bahi, 2013) and female (Torres et al., 2013) rats. In concert with these acute rewarding effects, Ibba et al. (2009) blocked ethanol-induced ERK phosphorylation (1 g/kg, 15 min) with a dopamine D1 receptor antagonist. The association between the rewarding effects of ethanol and accumbens dopamine release has also been linked to Ras-GRF2, an upstream effector of ERK signaling (Stacey et al., 2012). Increases in ERK phosphorylation post-alcohol appear to be maintained for a longer period of time in the amygdala (60–90 min) vs. accumbens and may mediate the anxiolytic and discriminative stimulus properties of alcohol exposure (Pandey et al., 2008; Besheer et al., 2012). In concert with these effects, Pandey and colleagues have elegantly described a functional role for a compromised BDNF-ERK-CREB signaling axis in the amygdala in the promotion of alcohol drinking (Pandey et al., 2006). They were able to demonstrate a reduction in ERK phosphorylation following antisense-mediated knockdown of the neuropeptide BDNF in three primary amygdala nuclei (basolateral, medial, and central). However, only a reduction of BDNF/pERK/cAMP response element-binding protein (pCREB) in the medial and central amygdala led to increased drinking, possibly as a means to restore functional signaling along this axis. This study demonstrates the ability of alcohol to alter nuclear and gene transcription systems downstream of ERK (also see Hansson et al., 2008). Indeed, Pandey and colleagues have hypothesized that compromised CREB function in the amygdala engenders an anxiety-like state that encourages drinking, and this theory has been supported in both rats and mice (Pandey et al., 2003, 2004, 2005; Zhang et al., 2011). Signaling along this axis has even been shown to mediate structural plasticity in the central and medial amygdala in response to alcohol drinking (Pandey et al., 2008). In comparison to rewarding doses of alcohol (~1.0–1.5 g/kg), acute administration of higher doses (2.5–4.5 g/kg) in rats produce reductions in neuronal ERK phosphorylation, and this may correspond to the aversive effects of higher doses (Anderson et al., 2010; Torres et al., 2013; Zhu et al., 2013) that tend to limit alcohol consumption in non-dependent drinkers. Interestingly, Hodge and colleagues reported that a lower dose of a MEK-ERK inhibitor given systemically increased alcohol drinking in mice while higher doses decreased drinking (Faccidomo et al., 2009), further suggesting that alcohol preference is directly related to neuronal ERK levels.

In addition to D1 dopamine receptors, a recent study described the role of brain peptide signaling, specifically the neuropeptide S (NPS)/NPS receptor system, in mediating the reinforcing effects of alcohol via ERK phosphorylation in the central amygdala (Thorsell et al., 2013). Typically, neuropeptides act through G protein-coupled receptors to activate several signal transduction cascades at once. However, Heilig and colleagues took advantage of the selective NPS receptor antagonist NCGC00185684, which preferentially blocks ERK phosphorylation over cAMP and calcium signaling. They demonstrated that NCGC00185684 reduced alcohol drinking and progressive ratio responding (a measure of motivation) for alcohol in non-dependent animals. Although this drug did not block reinstatement to alcohol-seeking behavior in rats, relapse may be more of a concern in dependent subjects seeking to continue along an abstinence regimen. In this regard, Ruggeri et al. (2010) found that amygdala NPS receptor expression is increased in withdrawn, alcohol-dependent rats, wherein NCGC00185684 might be expected to prove efficacious against relapse-like behaviors.

## Neuronal extracellular signal-regulated kinase (ERK) phosphorylation in the alcohol-dependent state

Indeed, mounting evidence suggests that ERK phosphorylation may play a unique role in alcohol dependence in addition to its effects in mediating the positive reinforcing effects of acute alcohol exposure. Importantly, the transition from recreational alcohol use to dependence is associated with an emergence of negative motivational symptomatology that promotes excessive drinking (Hansson et al., 2007; Gilpin and Koob, 2008). Such a condition is modeled via repeated cycles of alcohol intoxication and abstinence in rodents (Roberts et al., 1996, 2000), whereby intermittent exposure both represents the human alcoholic condition and is thought to expedite alcoholism-related plasticity (O’Dell et al., 2004; Breese et al., 2005). To measure the effects of excessive and repeated alcohol exposures on neuronal ERK phosphorylation, Sanna et al. (2002) employed a model of alcohol dependence that exposed rats to intermittent cycles (14 h or alcohol exposure, 10 h of abstinence) of dependence-inducing alcohol vapor (Gilpin et al., 2008). Chronic (14 d) vapor exposure reduced pERK levels at the end of the alcohol exposure period (i.e., at peak blood alcohol levels) in several brain regions including the accumbens and hippocampus (also see Roberto et al., 2003). In contrast, withdrawal (7–13 h) from this regimen significantly elevated ERK phosphorylation in the amygdala, at a time point commensurate with diminished blood alcohol levels and elevated operant alcohol self-administration (Edwards et al., 2012a). This neuroadaptation suggests that ERK-mediated signaling may mark and mediate plasticity induced by alcohol withdrawal in addition to the effects produced by drinking. Withdrawal-induced ERK phosphorylation is also in line with opponent process theories of drug addiction (Koob and Le Moal, 2008), whereby brain stress systems are hypothesized to be recruited and potentiated during abstinence from excessive drug exposure.

A remaining question is which neurotransmitters contribute to excessive amygdala ERK phosphorylation during withdrawal in alcohol-dependent animals. The neurobiological basis for the negative reinforcement mechanisms important for the development and maintenance of alcoholism may include neuroadaptations in central neuropeptide systems associated with stress and anxiety, including the amygdala CRF system (Timpl et al., 1998; Shekhar et al., 2005). Indeed, previous studies have demonstrated the recruitment of CRF in the amygdala as a critical element driving the excessive ethanol intake observed during both acute and protracted abstinence (Heilig and Koob, 2007). These studies implicated enhanced CRF signaling in discrete amygdala nuclei, including the central (Lack et al., 2005; Funk and Koob, 2007; Funk et al., 2007) and basolateral (Sommer et al., 2008) nuclei as neuroanatomical substrates driving ethanol dependence. Interestingly, intracerebroventricular administration of CRF *in vivo* increases ERK phosphorylation in the basolateral amygdala, but not central nucleus (Refojo et al., 2005).

In addition, given the remarkable persistence of the addicted state, some investigators have sought to focus on neuronal changes that last well into abstinence. Such a “post-dependent” state is associated with a stable elevation of forebrain MAPK signaling elements, including ERK kinases (Rimondini et al., 2002). As an extension of their preliminary studies, Heilig and colleagues (Hansson et al., 2008) sought to understand ERK cascade responsiveness to alcohol in the post-dependent state. Interestingly, ethanol-induced activation of MEK/ERK-associated pathways in the medial amygdala is lost in dependent animals. Moreover, ERK signaling is potentiated and suppresses the neuronal response to alcohol in the frontal cortex and nucleus accumbens. Both of these changes may possibly correspond to tolerance to the rewarding effects of alcohol in dependence, leading to an escalation of drinking if relapse occurs.

## Extracellular signal-regulated kinase (ERK) modulation of alcohol withdrawal and compulsive alcohol seeking

Cue-induced reinstatement of alcohol seeking activates ERK phosphorylation in the basolateral amygdala (Radwanska et al., 2008) and nucleus accumbens shell, and this is dependent on mGluR5 activation in both regions (Schroeder et al., 2008; Sinclair et al., 2012). These studies suggest that excessive ERK activity may correspond with potentiated glutamatergic transmission in alcohol dependence and relapse (Ron, 2004; Chandler et al., 2006; Szumlinski et al., 2007; Mason et al., 2009; Holmes et al., 2013).

ERK signaling would also appear to play a connecting role in the critical interplay between negative affective states (including anxiety and stress) and ethanol drinking (De Witte et al., 2003). Alcohol withdrawal potentiates anxiety-like behavior (Valdez et al., 2002) and sensitizes contextual fear memories (Bertotto et al., 2006) that in turn increase alcohol consumption (Bertotto et al., 2010). Interestingly, fear conditioning potentiates ERK phosphorylation in the basolateral amygdala during withdrawal in alcohol dependent animals (Bertotto et al., 2011). This effect was blocked by administration of MK-801, an antagonist of NMDA receptor channels, which are known to be dysregulated in the amygdala of dependent rats (Roberto et al., 2004, 2006). In a similar investigation, Gilpin and colleagues assessed neuronal activations patterns by measuring ERK phosphorylation following exposure to a discrete context previously paired with traumatic stress (bobcat urine), mimicking symptom provocation in human post-traumatic stress disorder (PTSD) patients (Edwards et al., 2013). In this study, a subgroup of rats that displayed a persistently high avoidance of trauma-related stimuli also exhibited a long-lasting post-stress escalation of alcohol drinking. High-avoidance, high-drinking rats also exhibited greater synchronicity between the dorsal prefrontal cortex and basolateral amygdala, as measured by individual within-subject and between-region correlations in ERK phosphorylation upon re-exposure to the traumatic stress-paired context. This finding corresponds to the regional synchronization found in PTSD patients upon symptom provocation (Gilboa et al., 2004), and may underlie the increased propensity for alcohol abuse in this population (Engdahl et al., 1998; Jacobsen et al., 2001). In addition, a heightened proclivity for high-risk prescription opioid use in PTSD sufferers (Seal et al., 2012) is one piece of evidence suggesting a common neurobiological substrate underlying alcohol and opioid use disorders in vulnerable populations.

## Effects of opioid exposure on neuronal extracellular signal-regulated kinase (ERK) phosphorylation

Endogenous opioids represent one of the body’s primary natural reward systems and play a central role in health and disease states (Bodnar, 2013), including drug addiction (Trigo et al., 2010). Exogenous opioids, ranging from opiate derivatives (e.g., morphine) to prescription opioid analgesics (e.g., oxycodone), interact with opioid receptors in the body to produce effects ranging from therapeutic to pathological (Shurman et al., 2010). Similar to alcohol, morphine (5 mg/kg, 20 min pretreatment) acutely elicits ERK phosphorylation in the rodent extended amygdala through a dopamine D1 receptor-dependent process (Valjent et al., 2004), while higher doses (10–50 mg/kg) produce no changes or even decreases in neuronal pERK levels, depending on brain region (Eitan et al., 2003; Muller and Unterwald, 2004; Valjent et al., 2004; Moron et al., 2010). In striatal neurons, mu-opioid receptor activation of the ERK cascade also appears to require receptor phosphorylation by G protein-coupled receptor kinase (GRK3) and arrestin3 recruitment (Macey et al., 2006). The rewarding effects of opioids, including oxycodone (Liu et al., 2009), are associated with ERK activity in the nucleus accumbens (Xu et al., 2012). With repeated exposure, psychomotor sensitization to morphine requires ERK phosphorylation in striatal D1 neurons (Borgkvist et al., 2008). Interestingly, genetic deletion of the ERK1 isoform leads to enhanced D1 signaling, greater phosphorylation of the ERK2 isoform, and potentiation of morphine reward (Mazzucchelli et al., 2002). In addition to the ventral striatum, Li et al. (2011) linked morphine reward to increases in central amygdala ERK phosphorylation downstream of NMDA receptor activation. A D1 receptor-dependent, ERK mediated process in the basolateral amygdala was also found to be required for opioid-related memory formation (Lyons et al., 2013). Interestingly, Laviolette and colleagues have provided evidence that opioid reward memory consolidation transitions from an ERK- and basolateral amygdala-dependent process to a CaMKII-dependent mechanism in the prefrontal cortex (Gholizadeh et al., 2013).

## Neuronal extracellular signal-regulated kinase (ERK) phosphorylation in the opioid-dependent state

Chronic heroin self-administration in rats produces a multitude of symptoms of dependence including elevated brain reward thresholds (Kenny et al., 2006) and a marked hyperalgesia that correlates with heroin intake (Edwards et al., 2012b). With extended access to heroin self-administration (≥6 h/day), animals will escalate their intake over time and begin to display signs of physical withdrawal either spontaneously or following mu-opioid receptor antagonism (Vendruscolo et al., 2011). Intake escalation represents a critical Diagnostic and Statistical Manual of Mental Disorders (DSM) criteria for dependence and is associated with multiple behavioral and neurochemical adaptations that persist or become manifest in protracted abstinence (Edwards and Koob, 2013).

In an attempt to shed light on brain regional neuroadaptations in ERK signaling following escalation of heroin self-administration, Self and colleagues (Edwards et al., 2009) examined ERK phosphorylation across eleven mesolimbic brain regions. Interestingly, naltrexone by itself significantly reduced ERK phosphorylation by 24–30% in heroin-naïve animals in both the ventral tegmental area and substantia nigra, suggesting a tonic activation of the ERK signaling cascade via endogenous mu-opioid receptor stimulation in these regions. This blunting of the ERK pathway by naltrexone was normalized in heroin self-administering animals receiving a naltrexone challenge, suggesting a relative potentiation of midbrain ERK signaling in heroin dependence. These data are in harmony with increases in total ERK1/2 levels found in the midbrain locus coeruleus in morphine-dependent rats (Ortiz et al., 1995). In forebrain regions, increases in pERK in the prefrontal cortex and hippocampus predominated following 24 h withdrawal either in the presence or absence of a naltrexone challenge. In comparison, naltrexone-precipitated withdrawal (vs. spontaneous withdrawal) facilitated greater increases in pERK in the nucleus accumbens and central amygdala. A similar pattern of accumbens ERK activation was found in morphine-dependent mice receiving a naloxone challenge, and did not generalize to the other MAPK-family members p38MAPK or c-Jun N-terminal kinase (JNK; Li et al., 2010). Extending these findings, Ciccarelli et al. (2013) delineated a role for ERK signaling in the regulation of epigenetic marking during opioid withdrawal. They found that naloxone-precipitated morphine withdrawal increased histone H3 phosphorylation through an ERK-dependent process in the nucleus accumbens. Such epigenetic changes at the histone level likely represent stable, long-term alterations in gene expression capacity (Robison and Nestler, 2011) that may underlie the persistence of addiction (Maze and Nestler, 2011). Recruitment of ERK signaling in the central amygdala was also found in morphine-dependent mice (Hofford et al., 2009). The central amygdala is critically involved in the expression of dysphoria and hyperalgesia that occurs during opioid dependence, a phenomenon mediated by CRF signaling (Heinrichs et al., 1995; McNally and Akil, 2002). Potentiated ERK signaling may enhance central amygdala CRF levels via CREB activity, which is increased in CRF neurons in morphine-dependent animals (Shaw-Lutchman et al., 2002).

## Extracellular signal-regulated kinase (ERK) signaling in the context of opioid withdrawal and relapse behaviors

Opioid withdrawal produces a long-lasting conditioned place aversion (CPA) in dependent animals (Stinus et al., 2000), indicative of the negative motivational state made manifest following dependence induction (Koob and Le Moal, 2008). Wang et al. (2012) recently demonstrated the importance of ERK-mediated epigenetic regulation within the ventromedial prefrontal cortex (vmPFC) in the extinction of aversive memories within the context of morphine withdrawal. In this study, re-exposure to a withdrawal-paired context elicited ERK phosphorylation and histone H3 acetylation in the vmPFC, while MEK-ERK inhibition prevented both induction of H3 acetylation and extinction of CPA. Thus, disruption of vmPFC ERK signaling may play a role in opioid craving and relapse under aversive conditions. In concert with this hypothesis, reduced levels of mu-opioid receptors and ERK signaling components were found in the prefrontal cortex of humans who suffered an opioid overdose (Ferrer-Alcón et al., 2004a,b).

A heightened propensity to relapse is one hallmark of opioid addiction that persists well into abstinence and is typically preceded by re-exposure to drug-associated cues and contexts (O’Brien, 1997). The motivating properties of such contextual cues to elicit relapse-like behavior has been modeled in rodents (Neisewander et al., 2000; Grimm et al., 2001), whereby alcohol (Bienkowski et al., 2004) and drug seeking actually increases with time in the abstinent state. For example, Shaham and colleagues demonstrated withdrawal time-dependent increases in baseline heroin seeking as well as footshock stress-induced reinstatement of heroin seeking (Shalev et al., 2001). This phenomenon has also been observed in classical (Pavlovian) conditioning models such as CPP. Expression of morphine CPP gradually increases over 2 weeks after the last drug-environment pairing (Li et al., 2008). Interestingly, re-exposure of animals to the morphine-paired environment at the latest withdrawal time point (14 days) increased ERK phosphorylation in the central but not basolateral amygdala. This was matched by similar increases in CREB phosphorylation, providing additional evidence linking this signaling axis in drug reward and addiction liability. Further, inhibition of central amygdala MEK-ERK activity with U0126 also prevented the expression of the magnified CPP in protracted abstinence.

## Conclusion and future challenges

Valjent et al. (2004) hypothesized that a strong activation of pERK across all three extended amygdala regions distinguishes drugs most likely to be abused from other psychoactive substances that are not considered to produce addiction (e.g., antidepressants, antipsychotics), in accordance with the suggested central role of these regions in dependence (Koob et al., 2014). Such a molecular litmus test may be useful to screen the potential addiction liability of trending recreational substances to complement traditional behavioral assays (Huang et al., 2012; Aarde et al., 2013). On a similar note, it is important to mention that the effect of ERK signaling in mediating compulsive drug seeking may generalize to natural rewards as well. Grimm et al. (2002, 2005) found that operant seeking of sucrose increases with withdrawal time, similar to artificial rewards. Following 3 weeks withdrawal, both cocaine- and sucrose-seeking behavior elicit striatal ERK phosphorylation, while only cocaine seeking increases protein kinase A (PKA) phosphorylation of AMPA GluR1 subunits (Edwards et al., 2011). Given the role of striatal endogenous opioids in signaling food palatability (Kelley et al., 2002), ERK activity in central reinforcement systems may play a critical neurobiological role in the global obesity epidemic (Potenza, 2014). With the extensive overlap in the neural circuitry engaged by both drug and food cues (Kelley et al., 2005; Volkow et al., 2013), further studies into the shared role of ERK signaling in addiction and obesity are warranted.

Ultimately, neuroscientists hope to produce medical breakthroughs from the basic science knowledge gleaned from a better understanding of altered ERK signaling in disease processes. Upon initial examination, the demonstrated role of ERK phosphorylation in mediating drug and alcohol dependence makes MEK/ERK blockade an attractive therapeutic strategy. Unfortunately, the ubiquity of MAPK/ERK activity in virtually all physiological processes makes systemic inhibition more poison than therapy (Balmanno and Cook, 2009). However, as discussed above in the study of Thorsell et al. (2013), directed activation or blockade of ERK signaling via target receptor ligands that are capable of selectively influencing this pathway represent one solution to this dilemma. In addition to NPS receptors, other promising targets of interest couple to ERK pathways, including CRF1 receptors (Punn et al., 2006; Kageyama et al., 2007; Hauger et al., 2009; Meng et al., 2011). In addition, Bruchas and Chavkin (2010) have proposed that targeting ligand-directed signaling at kappa-opioid receptors (KORs) may prove useful in the treatment of pain and addiction by selectively altering distinct members of the MAPK family (ERK, p38, and JNK). For example, while arrestin-dependent p38 MAPK activation appears to mediate aversion in animals, recruitment of JNK signaling in the absence of arrestin results in an inactivation of KOR activity. The same group demonstrated that repeated forced swim stress induces striatal ERK phosphorylation (Bruchas et al., 2008), similar to heroin withdrawal (Edwards et al., 2009). Stress-induced ERK activation was blocked by the long-lasting KOR antagonist norbinaltorphimine, a drug that has also been demonstrated to block escalation of heroin self-administration in rodents when injected systemically or into the ventral striatum (Schlosburg et al., 2013). These remarkable findings have propelled the design and development of new small molecule, functionally selective KOR antagonists (Zhou et al., 2013) that may prove efficacious in managing the hedonic dysregulation associated with drug and alcohol dependence (Koob and Le Moal, 1997; Kenny et al., 2006; Potter et al., 2011; Walker et al., 2012).

Finally, it will be critical to understand how ERK signaling interfaces with the multitude of other molecular signaling processes influenced by alcohol and opioids, ranging from classical kinase pathways to systemic inflammatory responses (Crews et al., 2011; Ting and Van Der Kooy, 2012; Ron and Messing, 2013). In addition, the interactive nature of and co-dependence on alcohol and opioids is a subject that has not been investigated at the level of neuronal ERK signaling. Alcohol and opioids activate a shared subset of neurobiological systems (Koob and Bloom, 1988; Herz, 1997; Koob et al., 2003; Siggins et al., 2003; Gianoulakis, 2009) and exhibit substantial similarity in terms of withdrawal symptomatology (West and Gossop, 1994; Edwards et al., 2012b). Thus, it is not surprising that existing studies have revealed a parallel recruitment of ERK signaling within stress and reward circuitry across important stages of the addiction timeline. The addiction field is currently challenged with the task of translating this basic knowledge at the signal transduction level into more effective treatments to curb excessive opioid and alcohol use. Continuing collaboration between addiction scientists and clinicians in the context of an important functional integration of addiction-related research aims at the National Institutes of Health is expected to drive these efforts in the coming decades.

## Authors and contributors

Eva R. Zamora-Martinez and Scott Edwards contributed to the conceptualization and writing of this review.

## Conflict of interest statement

The authors declare that the research was conducted in the absence of any commercial or financial relationships that could be construed as a potential conflict of interest.
